# Development and internal validation of a predictive score for the diagnosis of central adrenal insufficiency when morning cortisol is in the grey zone

**DOI:** 10.1007/s40618-022-01926-z

**Published:** 2022-09-26

**Authors:** F. Bioletto, A. M. Berton, E. Varaldo, D. Cuboni, C. Bona, M. Parasiliti-Caprino, N. Prencipe, E. Ghigo, S. Grottoli, M. Maccario, V. Gasco

**Affiliations:** Endocrinology, Diabetology and Metabolism; Department of Medical Sciences, University of Turin, Corso Dogliotti 14, 10126 Turin, Italy

**Keywords:** Central adrenal insufficiency, Hypopituitarism, Morning cortisol, Pre-test probability, Predictive score

## Abstract

**Background:**

When evaluating a patient for central adrenal insufficiency (CAI), there is a wide range of morning cortisol values for which no definite conclusion on hypothalamus–pituitary–adrenal (HPA) axis function can be drawn; in these cases, a stimulation test is required. Aim of this study was to develop an integrated model for CAI prediction when morning cortisol is in the grey zone, here defined as 40.0–160.0 μg/L.

**Methods:**

Overall, 119 patients with history of sellar tumour which underwent insulin tolerance test (ITT) for the evaluation of HPA axis were enrolled. Supervised regression techniques were used for model development.

**Results:**

An integrated predictive model was developed and internally validated, and showed a significantly better diagnostic performance than morning cortisol alone (AUC 0.811 vs 0.699, *p* = 0.003). A novel predictive score (CAI-score) was retrieved, on a 5.5-point scale, by considering morning cortisol (0 points if 130.1–160.0 μg/L, 1 point if 100.1–130.0 μg/L, 1.5 points if 70.1–100.0 μg/L, 2.5 points if 40.0–70.0 μg/L), other pituitary deficits (2 points if ≥ 3 deficits), and sex (1 point if male). A diagnostic algorithm integrating CAI-score and ITT was finally proposed, with an overall accuracy of 99%, and the possibility to avoid the execution of stimulation tests in 25% of patients.

**Conclusions:**

This was the first study that proposed an integrated score for the prediction of CAI when morning cortisol is in the grey zone. This score might be helpful to reduce the number of patients who need a stimulation test for the assessment of HPA axis function.

**Supplementary Information:**

The online version contains supplementary material available at 10.1007/s40618-022-01926-z.

## Introduction

Any disease that affects the pituitary gland can result in diminished secretion of one or more pituitary hormones. Central adrenal insufficiency (CAI) is characterized by inappropriately low ACTH secretion, leading to a failure in adrenal cortisol production [1–5]. Most signs and symptoms of adrenal insufficiency are not specific and chronically occur as fatigue, weight loss, nausea/vomiting, abdominal pain, postural hypotension, hyponatremia, and hyperkalemia [5–7]. An acute onset as adrenal crisis is also possible, and represents a life-threatening condition [8–10].

A prompt and correct diagnosis of CAI is mandatory, because adequate hormonal replacement therapy is lifesaving [4]. Formally, the gold-standard reference for this diagnosis would be represented by the dynamic assessment of hypothalamus–pituitary–adrenal (HPA) axis function in response to a stimulation test, with peak cortisol levels < 180 μg/L being indicative of CAI, whichever test is used [3, 4]. Insulin tolerance test (ITT) is often considered as the best reference, due to its high diagnostic accuracy to assess the entire HPA axis, reacting to a stressful hypoglycemia [3, 4, 11–16]; however, it requires close medical supervision and trained personnel, and it is contraindicated in patients older than 60 years, in those with history of seizures, or with documented or suspected coronary artery disease [17]. An alternative is represented by the ACTH test, performed either at low-dose (cosyntropin 1 µg) or at standard-dose (cosyntropin 250 µg) [3, 4, 11–16, 18, 19]; compared to ITT, the ACTH test presents less risks and contraindications, but still requires multiple blood samplings with an appropriate supervision by trained professionals [3, 4].

In light of these issues, the measurement of morning serum cortisol at 8–9 AM has been extensively studied as a practical screening test that would obviate the need for dynamic testing in an outpatient setting. Its evaluation is currently recommended as the first-line test by the Endocrine Society guidelines, which suggest values < 30 μg/L as indicative of CAI, while values > 150 μg/L as indicative of adrenal sufficiency [4]. Notably, however, the choice of these thresholds represents a weak recommendation based on very-low-quality evidence, and various other cutoffs have been proposed in literature so far. Among studies that used the ITT as gold-standard, the proposed cutoffs mostly spanned from 30 to 50 μg/L for the diagnosis of CAI [20–23], and from 103 to 170 μg/L for its exclusion [20–23]; among studies adopting the ACTH test as the reference test, the proposed cutoffs varied from 31 to 53 μg/L for the diagnosis of CAI [24–27], and from 88 to 136 μg/L for its exclusion [24–30].

Anyhow, despite this large effort to optimize the specific choice of the cutoffs to adopt, what clearly emerges from the available literature is that morning cortisol alone shows a quite poor performance in predicting HPA axis response to stimulation tests, except when it is particularly high or particularly low [4, 23]. In fact, there is always a wide “grey-zone”, in which morning cortisol levels are not by themselves sufficient to establish a definite diagnosis on the actual HPA axis function; in these cases, a dynamic assessment of cortisol secretion is still necessary and, up to date, unavoidable.

Considering the possible drawbacks and costs of performing HPA axis stimulation tests, an alternative and reliable approach for the diagnosis of CAI when morning cortisol levels are inconclusive would be highly desirable and useful in clinical practice; in fact, it would reduce the number of patients to be tested, therefore, preventing test-related risks and decreasing healthcare-related costs. Aim of this study was thus to create such a tool, by developing and internally validating a multivariable predictive scoring system that combined, through supervised regression techniques, morning cortisol levels with other potential predictors of CAI.

## Methods

### Patient selection

Data of all patients who underwent ITT for the evaluation of cortisol secretion at the Neuroendocrinology Clinic of our Center between January 2017 and April 2021 were collected from prospective registries and analysed retrospectively. ITT was performed by intravenous injection of 0.1–0.15 IU/kg of regular insulin at 0 min in normal weight and overweight/obese subjects, respectively; blood sampling for cortisol and glucose was performed every 15 min from 0 to + 90 min. After an overnight fast, the test began in the morning at 8.00–8.30, 30 min after a peripheral venous catheter had been placed into a forearm vein that was kept patent by slow infusion of isotonic saline.

The following inclusion criteria were applied: (a) history of pituitary disease with the indication to evaluate HPA axis function; (b) morning serum cortisol in the “grey zone”, here defined as 40.0–160.0 μg/L based on overall evidence from the available literature. The following exclusion criteria were applied: (a) incomplete ITT; (b) non-achievement of adequate hypoglycemia (glucose < 40 mg/dl); (c) pituitary diseases other than sellar masses. The choice to exclude patients with pituitary diseases other than sellar tumours (e.g., traumatic brain injury, pituitary hypoplasia, primary empty sella, etc.) was dictated by the aim to improve cohort homogeneity. No formal sample size calculation was done at the moment of study design; the sample size was determined on a pragmatic basis, dictated by the availability of eligible patients; all patients fulfilling the aforementioned eligibility criteria were included in the analysis.

Approval from the Ethics Committee of the City of Health and Science University Hospital of Turin was obtained for the analysis of patient data. Written informed consent was obtained from all included patients.

### Data collection

For each patient, all the following data were collected: age, sex, tumour type, tumour dimension, fasting glucose, nadir glucose at ITT, morning cortisol, peak cortisol at ITT, presence/absence of other pituitary deficiencies apart from CAI, previous pituitary surgery, previous pituitary radiation therapy (RT). Morning cortisol was measured the same day as the ITT, just before the beginning of the test. The diagnosis of other pituitary deficiencies was made according to current international guidelines [4].

### Analytical methods

Serum cortisol levels (μg/L; 1 μg/L = 2.759 nmol/L) were determined by a competitive electro-chemiluminescence immunoassay automated on Cobas e601 instrument (Roche Diagnostics GmbH, Germany). Analytical sensitivity was 0.18 μg/L. Intra- and inter-assay precision ranged from 3.0% to 5.7% and from 2.4% to 6.2%, respectively. Plasma glucose levels (mg/dL; 1 mg/dl = 0.056 mmol/L) were measured by gluco-oxidase colorimetric method (Glucofix, Menarini Diagnostici, Florence, Italy). All other biochemical variables were assayed in plasma or serum using standard methods.

### Statistical analysis

The study followed the TRIPOD statement for Transparent Reporting of a multivariable prediction model for Individual Prognosis or Diagnosis [31, 32].

Baseline patients’ characteristics were summarized using mean and standard deviation for continuous data and percent values for categorical data. Differences between groups were evaluated by one-way analysis of variance (ANOVA) or by Student *t* test for continuous variables, and by chi-squared test for categorical variables. Relevant predictors of CAI were first described through univariate logistic regressions; pairwise correlations between predictors were evaluated by Pearson correlation coefficient and reported in a correlation matrix; for all significant continuous predictors, the assumption of linearity with the log-odds of the outcome was visually assessed by locally weighted scatterplot smoothing (LOWESS). All variables were evaluated for inclusion in a multivariable logistic regression model using a backward selection. To simplify the clinical application of the model, morning cortisol values have been categorized in four equally spaced categories.

Model calibration was evaluated by the Hosmer–Lemeshow test. A tenfold cross-validation algorithm was adopted for internal validation, to provide an estimate of model performance on unseen data [33]. After a random split of the original sample into ten groups, the entire modelling process was repeated in nine of them, and its performance was evaluated in the tenth. The process was then repeated ten times, rotating the validation group at each round. Final model performance was obtained as the average performance over the ten iterations. To simplify the use of the model in clinical practice, a weighted risk score was created upon normalization and rounding of regression β-coefficients to the nearest integer value.

A cutoff of 0.05 was adopted for the definition of statistical significance. Statistical analysis was performed using STATA 17 (StataCorp, College Station, Texas, USA).

## Results

### General characteristics of the study population

**Fig. 1:**
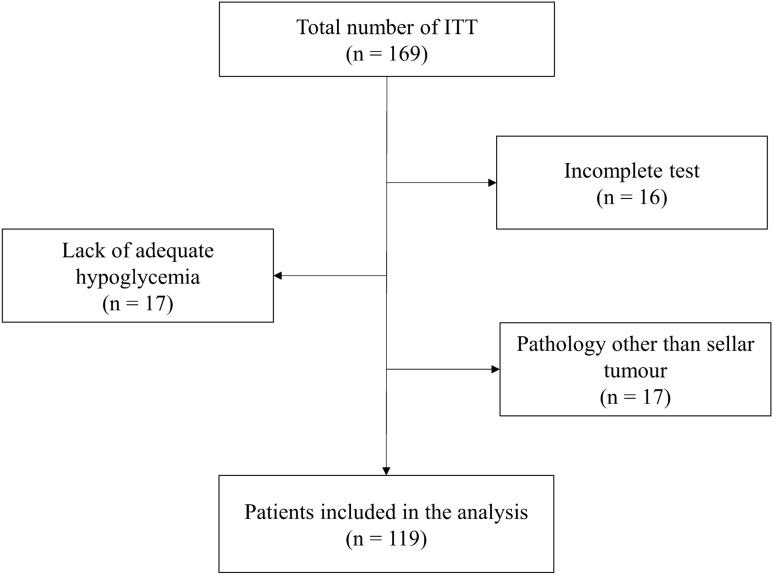
Study flow-chart. *ITT* insulin tolerance test

One-hundred and sixty-nine patients fulfilled the inclusion criteria and underwent ITT for cortisol assessment in our Center between January 2017 and April 2021. Of these, 16 were excluded, because the ITT was incomplete, 17 were excluded due to the non-achievement of adequate hypoglycemia (glucose < 40 mg/dl), and 17 were excluded because of a pituitary disease other than sellar tumour (Fig. 1). Thus, 119 patients were finally included in our analysis (35% female, mean age 42.7 ± 11.3 years). In 61 of these (51%), the cortisol peak during ITT was ≥ 180.0 μg/L; these patients were thus considered as having a normally functioning HPA axis. In the remaining 58 (49%), the cortisol peak during ITT was < 180.0 μg/L; these patients were thus considered as being affected by CAI.

### Model construction and internal validation

The association between relevant predictors and the outcome of interest was first explored through univariate logistic regression analysis (Table 1). Pairwise correlations between predictors were assessed by Pearson correlation coefficient (Supplementary Table 1). The appropriateness of a linear relationship between morning cortisol values and the log-odds of CAI was visually confirmed by LOWESS (Supplementary Fig. 1).

**Table 1:** Clinical characteristics of patients diagnosed with and without CAI, at univariate logistic regression analysis

Predictor	No CAI (n = 61)	CAI (n = 58)	OR	95%CI	*p* value
Age (years, mean ± SD)	44.0 ± 11.3	41.3 ± 11.2	0.98	0.95–1.01	0.194
Male sex (%)	51	79	3.71	1.65–8.34	0.002
BMI (kg/m^2^, mean ± SD)	26.5 ± 4.9	26.7 ± 4.4	1.01	0.92–1.10	0.909
Adenomatous tumour (%)	89	91	1.37	0.41–4.60	0.606
Tumour diameter (cm, mean ± SD)	2.5 ± 1.2	2.5 ± 1.0	1.04	0.74–1.45	0.837
Fasting glucose (mg/dl, mean ± SD)	83.2 ± 14.7	83.3 ± 8.3	1.00	0.97–1.03	0.995
Morning cortisol (μg/L, mean ± SD)	107.3 ± 28.2	86.1 ± 27.1	0.97	0.96–0.99	< 0.001
Morning cortisol category (%)					
130.1–160.0 μg/L	25	3	1.00	–	–
100.1–130.0 μg/L	33	29	6.38	1.27–31.92	0.024
70.1–100.0 μg/L	34	33	6.79	1.37–33.63	0.019
40.0–70.0 μg/L	8	35	30.00	5.10–176.34	< 0.001
Other pituitary deficits (%)					
None	48	40	1.00	–	–
One	25	21	1.01	0.40–2.57	0.986
Two	25	16	0.76	0.28–2.04	0.581
Three or more	3	24	8.83	1.82–42.83	0.007
Previous neurosurgery (%)	89	83	0.62	0.22–1.76	0.372
Previous radiation therapy (%)	16	35	2.68	1.13–6.39	0.026

*BMI* body mass index, *CAI* central adrenal insufficiency, *CI* confidence interval, *OR* odds ratio, *SD* standard deviation

**Fig. 2:**
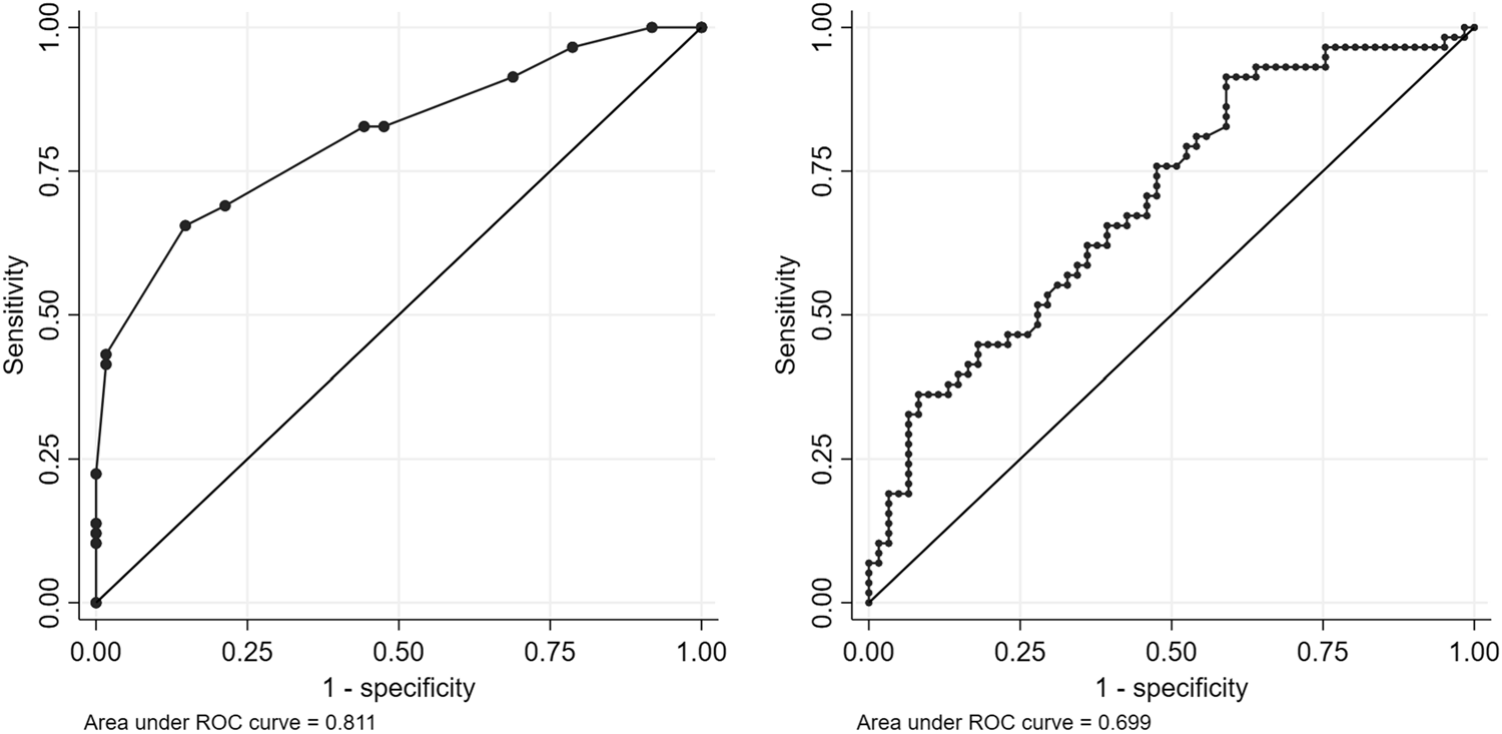
ROC curves of the multivariable model (left) and of morning cortisol (right) for the diagnosis of CAI. *CAI* central adrenal insufficiency, *ROC* receiver operating characteristic

All variables described at univariate analysis were considered for inclusion in a multivariable logistic regression; given the intention of developing a predictive score, morning cortisol was included in the model according to its described categorization. After a stepwise backward selection, the variables retaining statistical significance were morning cortisol categories (reference category if cortisol 130.1–160.0 μg/L; OR = 6.42, 95% CI 1.10–37.39 if cortisol 100.1–130.0 μg/L; OR = 12.65, 95% CI 2.14–74.91 if cortisol 70.1–100.0 μg/L; OR = 37.27, 95% CI 5.27–263.40 if cortisol 40.0–70.0 μg/L), ≥ 3 other pituitary deficits (OR = 17.81, 95% CI 2.19–144.92), and male sex (OR = 4.50, 95% CI 1.74–11.67) (Table 2). The predictive performance of the overall model was assessed by the calculation of the AUC at ROC analysis, which was equal to 0.811 (95% CI 0.729–0.877) (Fig. 2). This performance was significantly better (*p* = 0.003) than the one achievable by morning cortisol alone, which showed an AUC of 0.699 (95% CI 0.608–0.779) (Fig. 2).

**Table 2:** Prediction of CAI by multivariable logistic regression after stepwise backward selection of predictive variables; CAI-score point assignment according to multivariable regression coefficients

Predictor	OR	95%CI	*p* value	β-coefficient	Normalized coefficient	Points for CAI-score
Morning cortisol						
130.1–160.0 μg/L	1.00	–	–	–	–	0
100.1–130.0 μg/L	6.42	1.10–37.39	0.039	+ 1.859	$\frac{ + 1.859}{{ + 1.505}} = 1.235$	+ 1
70.1–100.0 μg/L	12.65	2.14–74.91	0.005	+ 2.538	$\frac{ + 2.538}{{1.505}} = 1.686$	+ 1.5
40.0–70.0 μg/L	37.27	5.27–263.40	< 0.001	+ 3.618	$\frac{ + 3.618}{{1.505}} = 2.404$	+ 2.5
Three or more other pituitary deficits	17.81	2.19–144.92	0.007	+ 2.880	$\frac{ + 2.880}{{ + 1.505}} = 1.914$	+ 2
Male sex	4.50	1.74–11.67	0.002	+ 1.505	$\frac{ + 1.505}{{ + 1.505}} = 1.000$	+ 1

*CAI* central adrenal insufficiency, *CI* confidence interval, *OR* odds ratio

The Hosmer–Lemeshow test did not reveal any significant miscalibration (*p* = 0.54). Internal validation of the model was performed through tenfold cross-validation, as already described. The final estimation of the model performance on unseen data, obtained as the average AUC over the ten iterations, was equal to 0.769, thus reassuring about a small overfitting effect.

### Score retrieval and risk class stratification

To simplify the use of the model in clinical practice, integer or half-integer point scores were assigned to each predictor upon normalization and rounding of regression β-coefficients, as reported in Table 3. According to the assigned coefficients, the retrieved score was structured on a 5.5-point scale. Due to its aim, this score will be referred to as CAI-score. Notably, this mild simplification did not lead to a relevant reduction in the predictive power of the model, since the AUC only slightly declined from 0.811 to 0.802 (95% CI 0.719–0.869).

**Table 3:** Cortisol peak at ITT and probability of CAI according to CAI-score

CAI-score	N of patients	Cortisol peak at ITT (μg/L, mean ± SD)	% of patients with CAI
0 points	5	249.5 ± 50.6	0%
0.5–1 points	19	208.8 ± 37.9	26%
1.5–2 points	42	197.3 ± 47.7	31%
2.5–3 points	28	183.3 ± 34.2	57%
3.5–4 points	17	142.4 ± 28.6	94%
≥ 4.5 points	8	125.6 ± 26.9	100%

*CAI* central adrenal insufficiency, *ITT* insulin tolerance test, *SD* standard deviation

**Fig. 3:**
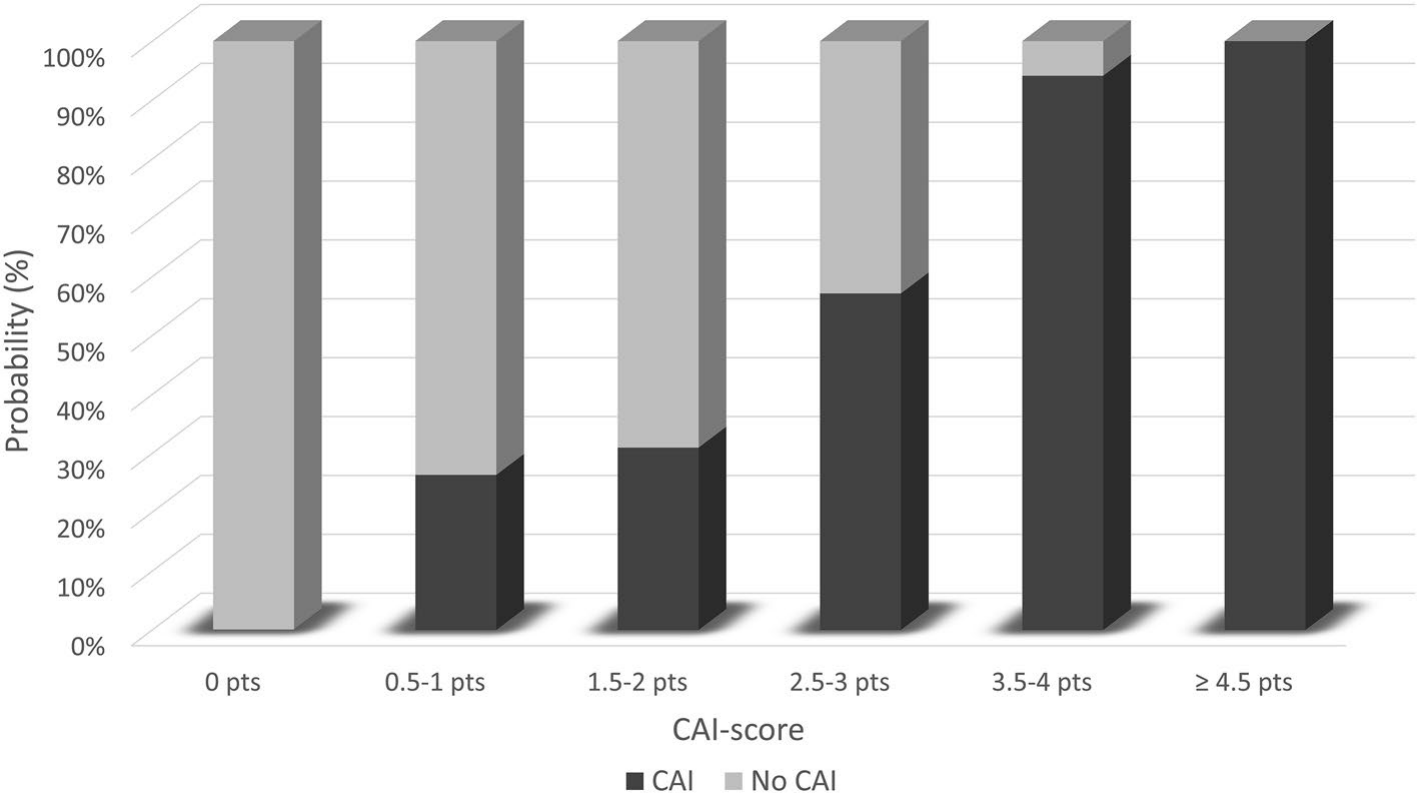
Probability of CAI according to CAI-score, graphically represented in a bar chart. *CAI* central adrenal insufficiency

**Fig. 4:**
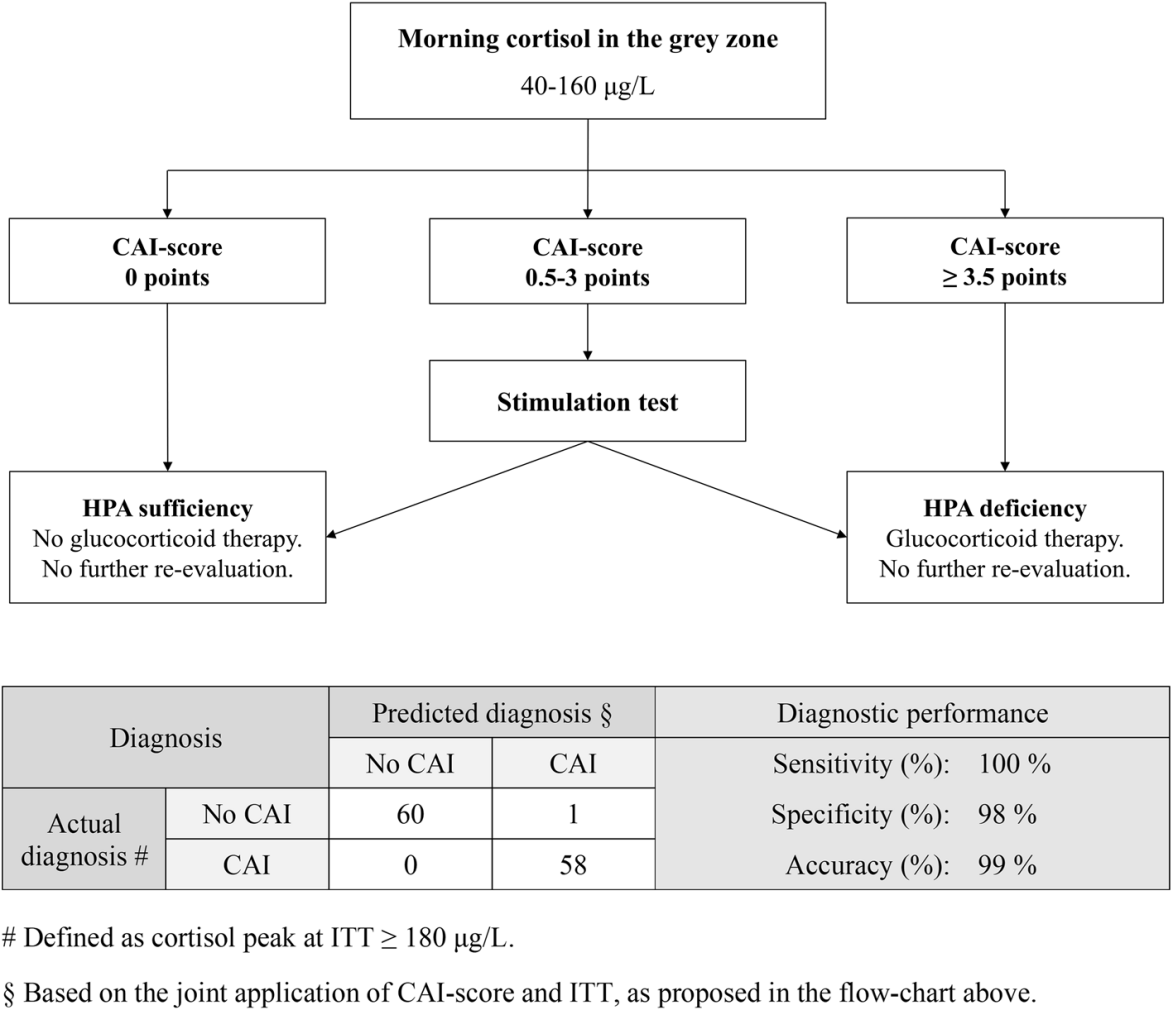
Proposed flow-chart for the diagnosis of CAI when morning cortisol is in the grey zone, based on the joint application of CAI-score and ITT (upper section). Contingency table with overall diagnostic performances of the proposed algorithm (lower section). *CAI* central adrenal insufficiency, *ITT* insulin tolerance test

Table 3 and Fig. 3 illustrate the stratification of patients according to CAI-score values. As it can be seen, CAI-score provides a good stratification of peak cortisol values at ITT (*p* < 0.001 at one-way ANOVA), and of the probability of CAI (*p* < 0.001 at chi-squared test). According to these results, we proposed a novel diagnostic algorithm for CAI, based on the integration of CAI-score and ITT (Fig. 4). The application of this algorithm would have avoided the execution of ITT in patients with a CAI-score = 0 (HPA sufficient) or with a CAI-score ≥ 3.5 (HPA deficient). An ITT would have still been necessary to correctly classify the remaining patients, i.e., those with a score comprised between 0.5 and 3 points. These cutoffs were chosen as a reasonable trade-off between diagnostic accuracy and the possibility to avoid stimulation tests. Overall, according to our data, the diagnostic accuracy provided by the proposed algorithm was near perfect (99%), with the only misclassification of 1 patient with a normal cortisol peak at ITT while having a CAI-score of 3.5. On the other hand, notably, it would have avoided the execution of ITT in approximately one-fourth (25%) of the patients in which morning cortisol values were ‘per se*’* non-diagnostic.

## Discussion

In this study, we developed and internally validated a multivariable model for the prediction of CAI when morning cortisol is in the grey zone. Our model showed a good predictive power for the discrimination between subjects with and without CAI, with an AUC of 0.811 at ROC analysis. Notably, this predictive performance was significantly better than the one achievable by morning cortisol alone, which showed an AUC of 0.699.

As previously discussed, morning cortisol alone is a highly imperfect predictor of CAI. According to a meta-analysis by Kazlauskaite et al. [20], CAI appears to be relatively uncommon when morning cortisol values are greater than 130 μg/L, while it is highly probable when morning cortisol values are less than 50 μg/L; however, these cutoffs do not provide full certainty in the diagnosis and, based on the individual patient data of the included studies, the presence of CAI could be demonstrated in patients with morning cortisol levels as high as 180 μg/L [20], while a normal HPA axis function could be observed in patients with morning cortisol levels as low as 30 μg/L [20]. Therefore, there is a wide span of values in which no definite conclusion about a correct function of HPA axis can be drawn. As a consequence, many patients with pituitary disease needs to be submitted to a stimulation test (either ITT or ACTH test) to exclude or confirm CAI. The present study proposed the integration of the information derived from morning cortisol levels with those obtainable by other possible predictive variables associated with CAI. In particular, according to our data, the predictive factors that remained statistically significant at multivariable analysis were the presence of ≥ 3 other pituitary deficits and male sex.

The association between the number of other pituitary deficiencies and the probability of CAI is not surprising; in fact, the global function of the hypothalamus and pituitary gland is deeply interconnected, and the coexistence of different pituitary deficits is a feature frequently encountered in hypopituitarism [34–38]. Moreover, our finding that CAI was significantly associated with the presence of ≥ 3 other pituitary deficiencies, and not with a lower compromise of the pituitary function, is coherent with the notion that the ACTH secretion is one of the most resistant among pituitary ones [34–38]. The role of sex as a risk factor for CAI is in line with previous findings by other authors [39–42]; male sex, in fact, has been found to be associated with a higher chance of hypopituitarism in various clinical settings, even after adjustment for other concurrent predictors [39–42]. The underlying pathophysiology has not been fully elucidated, but it is possibly related to the higher chance of aggressive behaviour of pituitary lesions in men [43–46], which might lead to a greater prevalence of hypopituitarism, both by a direct effect and by a more frequent need for aggressive treatments.

Notably, other factors potentially associated with CAI, such as previous neurosurgery and previous RT, were not included in the final model after the application of the stepwise backward variable selection. In particular, RT is known to be one of the most potent inducers of hypopituitarism, with a probability that increases with time; this was confirmed also by our data, as the association between RT and CAI was found to be statistically significant at univariate analysis. From a statistical point of view, the fact that it was excluded from the final multivariable model should be regarded as a consequence of that most of the information that it conveyed was already comprised, at least in our cohort, within the other included predictors. However, this is not fully surprising; in fact, after RT, also other pituitary deficiencies usually develop and morning cortisol values decrease, and, based on our results, these parameters appeared to be more sensitive than RT itself in predicting the presence/absence of CAI.

To the best of our knowledge, this was the first study that developed and internally validated an integrated score that combined multiple clinical and biochemical parameters for the prediction of CAI when morning cortisol is in the grey zone. Overall, it showed a better diagnostic performance than morning cortisol alone, and it thus may be of help in reducing the need for stimulation tests in patients that are evaluated for CAI; nevertheless, when considered alone, its diagnostic accuracy is still far from perfect, and a stimulation test would still be needed for patients having a CAI-score between 0.5 and 3 points. According to our data, this approach would have limited the number of patients needing a stimulation test by approximately one-fourth; this is a relative reduction, calculated over the number of patients with morning cortisol in the grey zone; in absolute terms, considering as denominator the total number of patients originally evaluated for CAI, the net benefit would have been lower, but cannot be computed based on the presented data. Another aspect to be discussed is the choice of the stimulation test; an important strength of our study was the use of the ITT for the definition of CAI, which is considered as the gold-standard test and gave a strong support to the reliability of our results. However, in clinical practice, the ACTH test is most often used. Compared to ITT, the ACTH test is less laboursome, less expensive, and associated with less side effects; therefore, the advantage of the application of the proposed score would be, in most cases, less marked.

Our study had some methodological limitations. First, it had a retrospective design; however, the retrieved data were prospectively collected and, most notably, the recall of baseline clinical features for each patient was based only on data retrieved from clinical reports preceding the beginning of any biochemical work-up by stimulation tests. Second, it has been only internally validated; an external validation of this score on a different patient cohort is still required before a definite assessment of its clinical utility can be made. Third, it was developed only for patients with a sellar mass; therefore, the retrieved results cannot be applied to patients with suspected CAI from other causes. Fourth, given the choice of using the ITT as the reference test for the diagnosis of CAI, the mean age of our cohort was relatively low; therefore, the use of the proposed score in older adults should be considered with caution, as this patient category was not adequately represented in its development.

In conclusion, this was the first study that proposed a predictive score for CAI when morning cortisol levels are in the grey zone; this was done by integrating—to the latter parameter—the information retrieved from other predictive factors associated with the outcome. Our final flow-chart represents a simple tool that could be adopted for a finer tailoring of the diagnostic process; this approach would limit the number of patients needing a stimulation test by approximately one-fourth while maintaining a near-perfect diagnostic accuracy.

## Supplementary Information

Below is the link to the electronic supplementary material.

Supplementary file1 (DOCX 92 KB)

## Data Availability

The data sets generated during and/or analyzed during the current study are not publicly available but are available from the corresponding author on reasonable request.
