# Supplementary material for: Development and internal validation of a predictive score for the diagnosis of central adrenal insufficiency when morning cortisol is in the grey zone
Source: J Endocrinol Invest. 2022 Sep 26;46(3):535–43. doi: 10.1007/s40618-022-01926-z (PMC9938019; doi:10.1007/s40618-022-01926-z)
Supplement: Supplementary file 1 — Supplementary file1 (DOCX 92 KB) [file 40618_2022_1926_MOESM1_ESM.docx]

**Supplementary Material**

**Supplementary Table 1.** Pairwise correlation matrix between predictors. Significant correlations are highlighted in **bold**.

|  | Age (years) | Male sex | BMI (kg/m^2^) | Adenomatous tumour | Tumour diameter (cm) | Fasting glucose (mg/dl) | Morning cortisol (μg/L) | N. of other pituitary deficits | Previous neurosurgery | Previous radiation therapy |
| --- | --- | --- | --- | --- | --- | --- | --- | --- | --- | --- |
| Age (years) | 1.000 | --- | --- | --- | --- | --- | --- | --- | --- | --- |
| Male sex | -0.007 | 1.000 | --- | --- | --- | --- | --- | --- | --- | --- |
| BMI (kg/m^2^) | 0.183 | **0.244** | 1.000 | --- | --- | --- | --- | --- | --- | --- |
| Adenomatous tumour | -0.005 | 0.045 | -0.069 | 1.000 | --- | --- | --- | --- | --- | --- |
| Tumour diameter (cm) | 0.031 | **0.331** | 0.174 | 0.119 | 1.000 | --- | --- | --- | --- | --- |
| Fasting glucose (mg/dl) | **0.289** | -0.114 | 0.078 | 0.065 | -0.028 | 1.000 | --- | --- | --- | --- |
| Morning cortisol (μg/L) | 0.047 | -0.058 | -0.025 | -0.080 | 0.111 | 0.077 | 1.000 | --- | --- | --- |
| N. of other pituitary deficits | 0.146 | **0.188** | 0.299 | **-0.190** | 0.145 | -0.029 | -0.041 | 1.000 | --- | --- |
| Previous neurosurgery | 0.126 | **0.201** | -0.068 | 0.103 | **0.190** | -0.036 | 0.091 | 0.168 | 1.000 | --- |
| Previous radiation therapy | -0.153 | **0.186** | -0.043 | **0.194** | -0.044 | 0.171 | **-0.218** | 0.118 | **0.237** | 1.000 |

Abbreviations: BMI, body mass index; N, number.

**Supplementary Figure 1.** Visual assessment by LOWESS of the linearity assumption between morning cortisol values and the log-odds of CAI.


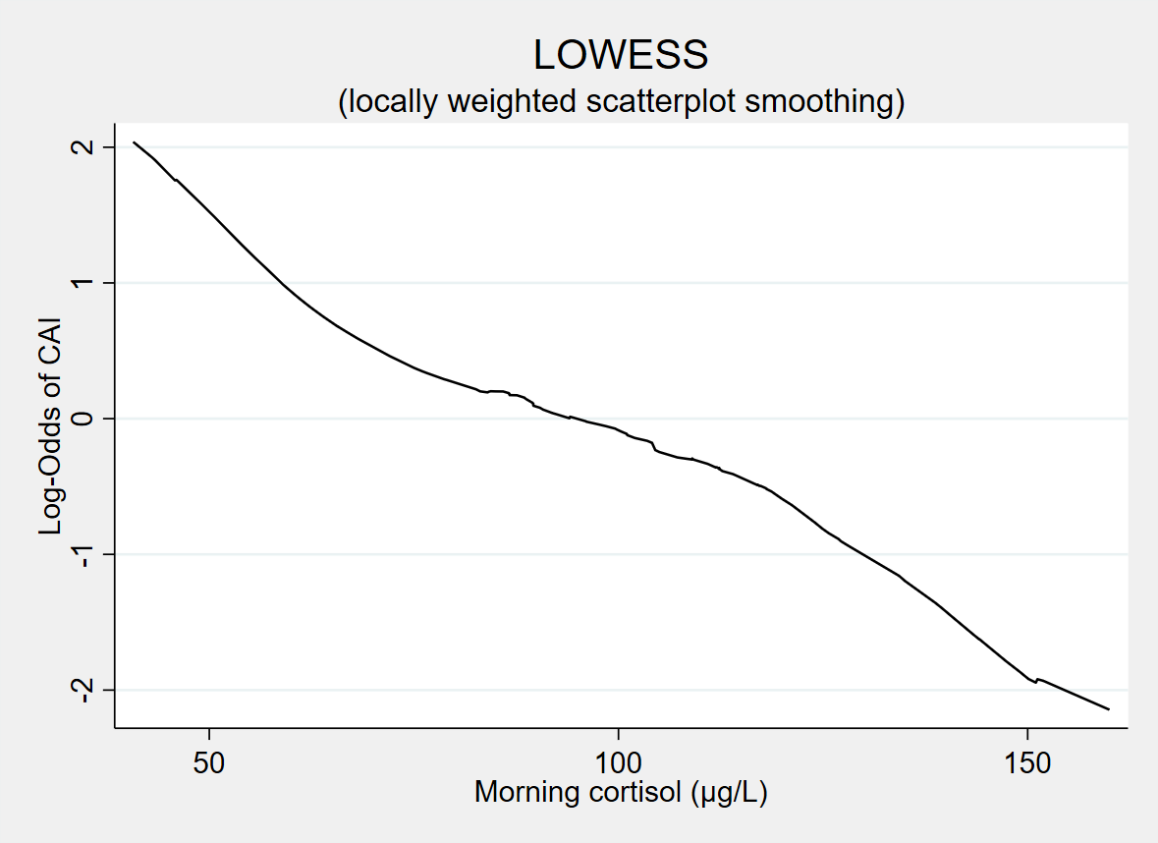


Abbreviations: CAI, central adrenal insufficiency; LOWESS, locally weighted scatterplot smoothing.
